# Supplementary figures and images for: Study on the effect of front retaining walls on the thermal structure and outflow temperature of reservoirs
Source: PLoS One. 2021 Dec 9;16(12):e0260779. doi: 10.1371/journal.pone.0260779 (PMC8659636; doi:10.1371/journal.pone.0260779)

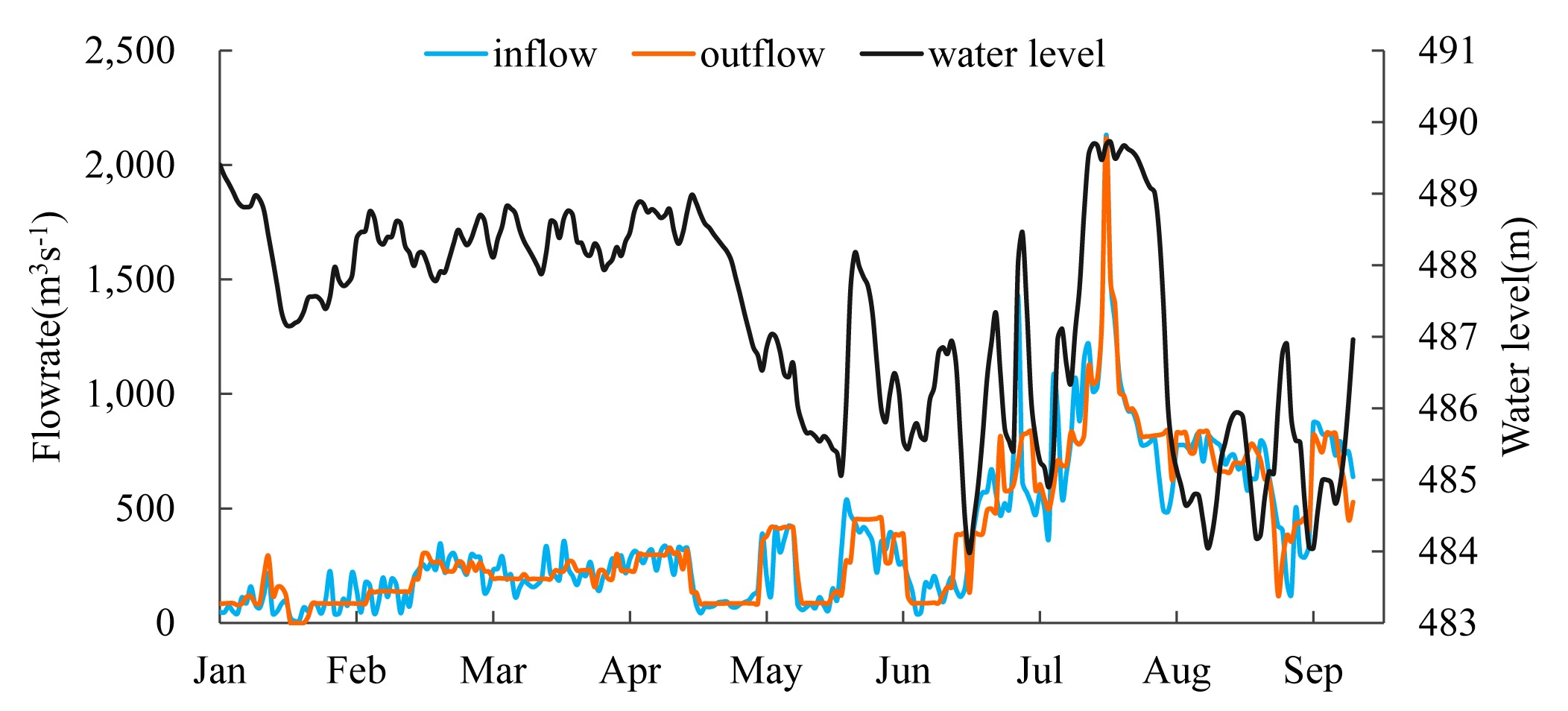

Supplement: S1 Fig — (TIF) [file pone.0260779.s001.tif]
