# Supplementary material for: Study on the effect of front retaining walls on the thermal structure and outflow temperature of reservoirs
Source: PLoS One. 2021 Dec 9;16(12):e0260779. doi: 10.1371/journal.pone.0260779 (PMC8659636; doi:10.1371/journal.pone.0260779)
Supplement: Attachment — Submitted filename: Response to Reviewers.docx [file pone.0260779.s002.docx]

# Response to Reviewers

Dear reviewer,

We are grateful for the constructive comments and suggestions. Based on your comments, we have revised the relevant content in the manuscript.

Firstly, we improved the quality of the introduction including revising the lumped citation, summarizing the results of some academic papers, and further refining and highlighting the innovation of our paper. Secondly, we professionalized the language of the paper, and explained the reason why the dimensions and the specific models were used. Secondly, the figures and formats of the paper were modified to meet the publishing requirements of your journal.

Our point-by-point responses to your comments are provided below.

***1): In the introduction section avoid using lumped citation and discuss the cited papers.***

Response to reviewer’s comment No. 1:

Thank you so much for your constructive suggestion.

Based on your suggestion, we made major revisions on Introduction. Firstly, the front retaining wall is a new selective withdrawal facility, thus we primarily reorganized the logical structure about how to elicit this research object and emphasized more its advantages to the engineering, such as “A front retaining wall (FRW) may be a more suitable choice which can meet the needs of the easy operation and low cost.”

Secondly, we almost rewrote the citation about previous research results of selective withdrawal systems and facilities by reintegrating literature cited. This mainly exists in the fourth paragraph that is as following:

“Many scholars have drawn a conclusion that selective withdrawal is the most effective means of controlling the quality of water released downstream. Deeper withdrawals also facilitate heat transfer in the water column, increase the thickness of the metalimnion and weaken the stratification stability. Through experimental study and numerical simulation, TCCs with different water-retaining proportions have been found to affect the flow field and thermal characteristic in reservoirs. A top-TCC is recommended for a smaller thermal stability or a cool outflow in the summer. Stop-log gates can mitigate the cold temperature effect in spring and summer. Moreover, stoplog gates can influence the flow patterns including the flow regime and velocity distribution at the intake and the flow velocity at the trash rack section varies vertically in a great manner. Thus, for distinct water management requirements, both relevant selective withdrawal systems and corresponding thermal properties may have a certain gap. As a new engineering measure, an FRW is mainly designed for the controlling of water released downstream. Tuo et al. illustrated the remained old dam dismantled above 240.2 m asl, standing before the new dam and acting as an FRW in Fengman Reservoir (in northeast China), can strengthen the flow motion in the surface layer and lower the outflow temperature in winter.”

Finally, we refined the expression of the article, such as “Water Temperature is an essential environmental factor of reservoirs which can affect water quality, nutrients distribution and even ecosystem” and “Thus, research on the evolution law of the stratified thermal state and discharged water temperature is the basis for optimizing the allocation of reservoir water.”

***2):*** ***Justify why this is a 2D problem rather than a 3D one.***

Response to reviewer’s comment No. 2:

I think your question is very interesting and worthy of inquiry. It runs through the entire water temperature research and continues to develop until now.

When it comes to a three-dimensional problem, the other dimensions will involve the width direction of reservoirs and the sectional vertical direction in front of the intake other than the vertical and longitudinal direction of reservoirs.

Firstly, for the width direction of the reservoir, the distribution of water temperature shows a slight change. The phenomena are common in many reservoirs especially in the shallow but long reservoir. For example, Tuo et al carried out the field observation before the intake in the Fengman Reservoir (in the northeastern China) to investigate three-dimensional effect [1]. The observation spots are shown in Fig 1 and comparisons of water temperature in heating period and cooling period are shown in Fig 2. We can see that the water temperature structure at the four perpendiculars in each period is basically the same, indicating that there is no significant change in the cross section and longitudinal flow direction of the water temperature in front of the dam.

Another reservoir in China, Ertan Reservoir, also conducted similar monitoring, which proved the existence of this phenomenon [2]. Fig 3 shows comparison of water temperature distribution on the three vertical lines in front of the dam. The surface temperature on the three vertical lines from left to right are 21.5℃, 21.5℃ and 21.1℃ respectively. The bottom water temperature is 10.1℃. Thus, there is basically no change in the water temperature for the width direction. Both cases can verify the weak and negligible differences in the width direction of reservoirs.

**Fig 1. Observation spots of vertical water temperature in front of the Fengman dam**

**Fig 2. Comparison of water temperature in heating period and cooling period**

**Fig 3. Comparison of water temperature distribution in front of the dam**

Secondly, water usually flows towards a sink of quite small dimensions. Thus, for the local vertical direction in front of the intake, local mixing including the drawdown and vortex caused by the retaining wall is relatively tiny. Fig 4 shows the discharge strong enough to overcome buoyancy forces both in the hypolimnion and at the thermocline [3]. We can see the drawdown into a surface outlet whose motion may be modeled as flow into a plane wall. The condition is similar to our selective withdrawal condition (Fig 5). The drawdown exists only a small range near the offtake which influences less on the overall temperature distribution profile. Moreover, we pay more attention to the water temperature variation in the vertical and longitudinal directions of the reservoir. The range of the vortex is small, and its impact on the water temperature is a very complex problem that requires a three-dimensional model to simulate. But this is not the focus of this paper, and will not have a fundamental impact on the results of this paper.

**Fig 4. Discharge strong enough to overcome buoyancy forces both in the hypolimnion and at the thermocline**

**Fig 5. The local flow field distribution in front of the dam in July with a retaining wall**

In conclusion, the slight change in the two directions can be ignored when mathematical simulation is performed. Thus, it can be calculated as a two-dimensional problem.

**Reference:**

1. Tuo Y.C., Liu Z.G., Deng Y., Li J., Liang R.F., Li N., 2014. Water Temperature of the Fengman Reservoir with Seasonal Ice Cover. Progress in Water Science. (05),731-738.
2. Li B.D., Li J., Li K.F., Deng Y., 2009. Field Observation and Analysis of Water Temperature Distribution in Front of Dam and in Outflow in Ertan Reservoir. Progress in Water Conservancy and Hydropower Science and Technology. 29(04):21-23.
3. Fischer H. B., List E. J., Koh R., Imberger J., & Brooks N. H.,1979. Mixing in Inland and Coastal Waters. Academic Pr. 390-392 pp.

***3):*** ***Enhance the quality of the figures.***

Response to reviewer’s comment No. 3:

Thank you for your suggestion. We complemented Fig 9 with corresponding legends, and improved the accuracy from 300dpi to 500dpi of all the chart with Adobe Illustrator CC software. And then we upload our figure files to the Preflight Analysis and Conversion Engine (PACE) digital diagnostic tool to ensure that figures meet PLOS requirements.

***4):*** ***Add nomenclature to the paper to enhance readability.***

Response to reviewer’s comment No. 4:

Thank you so much for your comment. We have turned some vernacular texts into nomenclature, such as the phases from “the upper warmer water layer” to “epilimnion”, from “the bottom stratosphere” to “hypolimnion”, from “withdrawal layer” to “mean plug flow” and so on.

In addition, we reorganized the whole paper, deleted redundant terms and modified the grammar of the paper to increase the refinement of the paper. Moreover, we submitted our paper to AJE again for polishing, and the polishing certificate is as follows.

***5): Explain why CE-QUAL-W2 model is hired.***

Response to reviewer’s comment No. 4:

Thank you so much for your constructive comment. We listed the reasons for using CE-QUAL-W2 and added them to our paper in lines 107-113 and cited relevant references. The specific reasons are as follows:

Firstly, in this paper, an unsteady two-dimensional model (along longitudinal and vertical directions) is the minimum requirement to study spatial-temporal thermal regime variations in the Dongqing Reservoir (Fig 1), which is a long-but-narrow reservoir with a longitudinal gradient in temperature that is not small enough to be neglected. Consequently, we conducted the present study by using CE-QUAL-W2, a two-dimensional laterally averaged hydrodynamic and water quality model. This model is mainly applied to water bodies with longitudinal and vertical temperature gradients.

Secondly, the critical model parameters of this model involve many factors including the longitudinal eddy viscosity coefficient, longitudinal eddy diffusion coefficient, wind shielding coefficient, extinction for pure water, solar radiation absorbed in the surface layer and dynamic shading coefficient and so on. These settings ensure the accuracy of the model. In this paper, the average MAE of the vertical water temperature in front of the dam and outflow temperature are 0.46°C and 0.23°C respectively, which are both within the error limit.

Finally, it provides accurate simulations of the stratification and density currents and has been widely used to study various reservoirs and river impoundments worldwide [4,5]. More importantly, it can meet the need of adding the front retaining wall when it comes to selective withdrawal.

In conclusion, From the aspects of dimensions, accuracy, application scope and engineering needs, it can achieve the simulation we want.

**Reference：**

4 Lee R., Biggs T., Fang X., 2018. Thermal and Hydrodynamic Changes under a Warmer Climate in a Variably Stratified Hypereutrophic Reservoir. Water. 10(9), 1284. 4.
5 Chung SW, Oh JK., 2006. Calibration of CE-QUAL-W2 for a monomictic reservoir in a monsoon climate area. Water Sci Technol. 54(11-12), 29-37.

***6): Clearly justify the mismatch in the verification case.***

Response to reviewer’s comment No. 4:

Thank you so much for your constructive comment. We listed the reasons why the mismatch exists in lines 203-209. The specific reasons are as follows:

Firstly, the deviation was partly due to the meteorological condition. Our data originated from the Zhenfeng Meteorological Station which was about 40 km away from the dam site. The wind speed and direction had certain deviations from the wind conditions in the open area of the reservoir area. Though we calibrated the wind parameters, there may still be a certain deviation in wind direction and speed.

Secondly, diurnal variations of water temperature also take effect. Related research indicates that the reservoir would have a more subtle mixing process within a day [6-8]. The epilimnion is heated during the daytime and cooled at night and subject to the influence of temperature fluctuations every day and night, resulting in diurnal variations. However, our meteorological data is given a fixed value each day, which has a certain error from the actual situation.

Finally, the outflow daily regulation of the reservoir will generate internal waves, leading to changes in the thickness of the epilimnion and the thermocline, and then affecting thermal structure in front of the dam [9]. As a reservoir with power generation demand, the generation of internal waves in Dongqing Reservoir makes the simulation of water temperature inaccurate. The internal waves are closely related to the flow process. The larger the flow is, the greater the internal wave is. The flow from July to August is larger compared with others. Larger and stronger internal waves cause greater errors, and these errors are not considered by our model. Therefore, the MAE in July and August is 0.86℃ and 0.71℃ respectively, while the average MAE is 0.45℃.

However, these errors are within a reasonable range with a relative error of 4.4% and 3.5% in July and August considerably.

**Reference：**

6 Yang Y, Deng Y, Tuo Y, Li J, He T, Chen M., 2020. Study of the thermal regime of a reservoir on the Qinghai-Tibetan Plateau, China. PLoS ONE 15(12): e0243198.
7 Vercauteren N, Huwald H, Bou Zeid E, Selker JS, Lemmin U, et al., 2011. Evolution of superficial lake water temperature profile under diurnal radiative forcing. Water resources research 47.
8 MacIntyre S, Romero JR, Kling GW (2002) Spatial-temporal variability in surface layer deepening and lateral advection in an embayment of Lake Victoria, East Africa. Limnology and oceanography 47: 656–671.
9 Xie Q, Liu Z, Chen Y, et al., 2019. Internal waves in a deep run-of-river reservoir under hydro-peaking. Journal of Hydroelectric Engineering, 38(1):41-51. (in Chinese)

Once again, thank you for your valuable suggestions and comments that enable us to substantially improve this paper. We hope the corrections will be accepted.

Best wishes,

Corresponding Author: Associate Prof. Youcai, Tuo

2021-8-6
